# Trench Mouth

**Published:** 1917-10

**Authors:** F. M. Wells

**Affiliations:** Research Officer, C.A.D.C.


### TRENCH MOUTH

MAJOR F. M. WELLS, RESEARCH OFFICER, C.A.D.C.

The name of "Trench Mouth," which has been given to this ulcerative condition of the throat and mouth, has become the source of misunderstanding which has not been dissipated by the labors of a few writers who have been called upon to furnish the results of their knowledge upon this subject. This is one of the numerous examples of the magic influence of a word.

I have had occasion to study this affliction under all its forms, during the course of the last seven months, and have been able to determine that it is separated by special properties from some other inflammations, accompanied with membraneous exudations—afflictions which are very distinct, and the characters of which I will point out in another report which will follow the present.

### ETIOLOGY—I

The appearance of this affection in the mouth varies very much according to its extent, or to its greater or less duration. It generally presents itself after a sore throat under the form of a grayish ulceration, occuyping the undulating border of the gums; the tartar is deposited in greater quantity that in the healthy state on the surface of the teeth as the disease progresses. Their line of insertion is more particularly the seat of the disease, so that the adhesion of the gum to the neck of the tooth being gradually destroyed, a looseness of the teeth is the result. Frequently the affection extends to the mucous membrane, lining of the lips, and more often, the cheeks; a white patch arises at the point of contact. It soon increases and becomes gray; sometimes it sinks deeply and the edges of this foul ulcer are swollen and a livid red color. Thick patches are detached from its surface and are replaced by new layers. The breath is rendered intolerably offensive as the disease progresses, and the disease then assumes the most specious resemblance to the true gangrene of the mouth, which is a more dangerous affection and one of an entirely different nature. I purposely omit peculiarities, the details of which at present would be out of place, and which can only be observed in following the progress and the successive stages of the disease. This variety of stomatitis is very infective. It is not selective as to sex, social conditions or occupation. Previous diseases have all to do with the production of this variety, especially if they are of that type which cause a viscous secretion from the salivary glands, causing a rapid fermentation and propagation of spirochaetes and fusiform bacilli.

### BACTERIOLOGY

In this variety of ulcerative stomatitis it is found microscopically that a variety of organisms are present. Vincent's (spirochaetes and fusiform bacilli) being the predominating ones in the smear from the mouth.

### DIAGNOSIS

The diagnosis is made on the character of the outset of the disease, and is usually found to be the progressive invasion of the disease after a sore throat. The mucous membrane in the back part of the mouth and distant part of gums back of third molar being first involved by mucous patches, the line of insertion of the lower anterior teeth being next involved. In the early stages of the disease the saliva is viscous and causes rapid fermentation in the mouth, as it is held in suspension on the mucous membrane and between the teeth. In early stages of the disease, the tongue has a morbid coating.

### SYMPTOMATOLOGY

At the outset of this variety of pyorrhea the patient can usually recall having suffered with a sore throat and can very often give a positive date of first manifestations. Mouth and teeth are involved soon after, and if the patient is questioned it is learned that in the beginning of the disease these attacks were very mild, but they have gradually increased as the conditions became worse. The teeth at the beginning of exacerbation are not painful when brought into occlusion, but as the attack progresses they feel elongated and are loose and painful when brought together.

### TREATMENT

Within twenty-four hours, using one teaspoonful Fowler's solution every four hours, the painful symptoms gradually subside, the gums and teeth feeling quite comfortable after the second day.

If a patient presents himself with his mouth in a painful condition when teeth are brought into occlusion and feel elongated, the hygienic treatment in this variety is very important. If the disease is characterized by the presence of very little pus, the first step in the treatment is to get the patient to rinse out the mouth thoroughly with a warm, mild saline solution, repeating several times to remove as much mucous as possible from between the teeth and mucous membrane; then immediately follow with about a teaspoonful of Liq. Arsenicalis, instructing the patient to rinse it well over and hold it in the mouth for a minute or so, at same time cautioning him not to swallow any. This treatment should be carried on for two days, three times a day, and the following four days, twice daily. Several patients can be treated at the same time, as they do not require to be placed in chair for such treatment.

This variety of pyorrhea is invariably accompanied by catarrh in the nose, and to get quick results in the mouth, instruct the patient to use a mild saline solution three or four times per day and clean the nose out thoroughly and keep perfectly clean. On the second or third day of treatment, if the teeth are no longer painful, thorough prophylaxis on the part of the patient and the dentist is necessary, but such scaling should not be done until mouth feels comfortable. Treatment should be continued for some time to prevent recurrence. For this purpose, an issue of a Dentifrice, containing 5 per cent. Liq. Arsenicalis, should be given to each man who presents himself for treatment. I have already supplied over 1,500 cakes to men and at the present time I am trying to get an order through for 100,000 for immediate distribution to Dental Clinics.—*Oral Health.*

